# Munyon's "Remedies"

**Published:** 1898-06-04

**Authors:** 


### The Hospital

A Journal of

The Medical Sciences and Hospital Administration.

Vol XXIV.—No. 610.] Edited by Sir Henry Burdett, K.C.B., and Solomon C. Smith, M.D., M.R.C.P. [June 4, 1898.

### Munyon's "Remedies."

The General Medical Council, at its session on Thursday last, received some curious and interesting information with regard to the manner in which the proceedings of a notorious quack are conducted, and also, indirectly, with regard to the credulity of the public. Certain newspapers have lately given much prominence to "Professor" Munyon's announcements of the invaluable qualities of his so-called "cures"; and these announcements have been adorned, in many instances, with an illustration supposed to represent the "Professor" himself. A casual glance at the advertisements shows that the "cures," which we take to be an American synonym for "remedies," are numerous; and that they are distinguished from one another as being "heart-cure," "gout-cure," or "liver-cure," and so on through a long list of the organs of which the human body is composed, and of the ills to which it is heir. The Incorporated Medical Practitioners' Association had the unkindness to complain to the General Medical Council that a Mr. Joshua Hamilton Hart, who was on the Medical Register as a Licentiate of the Royal Colleges of Physicians and Surgeons of Edinburgh, and of the Faculty of Physicians and Surgeons of Glasgow, and whose registered address was Cliff Cottage, Morley, near Leeds, had "associated himself with, and was a party to, the sale and dissemination and advertisement of these quack remedies, and that he acted as consulting physician at the place where such quack remedies were administered and sold, and permitted his name and qualifications to be advertised as those of the consulting physician at such place." Mr. Hart was put into the witness-box by his counsel; and the strangers' gallery during the hearing was adorned by the presence of "Professor" Munyon himself, who thus afforded to spectators an opportunity of judging of the accuracy of the published portraits. It appeared from Mr. Hart's own statement that he became connected with the "Professor" through the medium of an advertisement, which set forth that the services of a registered medical practitioner were required, that a homœopath would be preferred, and that application was to be made at a given address. The "Professor," apparently, does not belong to a school of philosophy which despises comfort, or even luxury, for Mr. Hart found him at the Hotel Cecil, and entered into his service for a fixed remuneration, the amount of which was not stated, but which, judging from the appearance of the recipient, was not excessive. The duties of the "Consulting Physician" to the "Munyon Company" were to attend daily at the office in Shaftesbury Avenue, to peruse letters from applicants for treatment, and to endorse each letter with the name of a disease, or of some organ which he considered to be the seat of disease. The letters so endorsed were sent to the packing department, and there dealt with in such a manner that an applicant whose letter was endorsed "lung" would receive a packet containing "lung-cure"; one, whose letter was endorsed "liver" would receive a packet containing "liver-cure"; and so forth through a long anatomical and pathological catalogue. Mr. Hart admitted to the Council that even his diagnostic powers were sometimes overtaxed by the descriptions of ailments which he received; and in such cases his practice was to use the endorsement "too vague," which meant that the applicant would receive a form of inquiries to be filled up. In some other instances, in which the symptoms described by the sick person might possibly admit of more than one interpretation, Mr. Hart was accustomed to use the word "nerves" as a secondary or supplementary endorsement. When he did this the applicant received a double supply of medicine, a packet of "nerve cure," in addition to the "gout cure" or "liver cure," which was furnished as a remedy for the primary indications of his case. Mr. Hart farther admitted that he did not know what any of the "cures" contained, that he did not know whether they were all alike or not, and that he did not know whether any active ingredient entered into the composition of any of them. He explained the letters and stops, "M.B., M.A.," which followed his signature to a letter, and which were followed by "L.R.C.P.E., L.R.C.S.E., and L.F.P.S.G.," as meaning "Member of the British Medical Association," and, on being asked if he were indeed a member, admitted that he had not recently paid his subscription. He did not know of what science or branch of knowledge Munyon was a "professor," or whether he had received any medical education. In these circumstances it is not surprising that the Council adjudged Mr. Hart to have been guilty of "infamous conduct in a professional respect," and ordered the erasure of his name from the Medical Register. He was only employed, as far as could be ascertained, because the Munyon advertisements assert that the selection and distribution of the "cures" is controlled by a duly qualified and registered practitioner. The public ought to feel greatly indebted to the Medical Practitioners' Association and to Dr. Hugh Woods, its President, who conducted the case with much ability, for effecting an exposure which, if there were any limits to human credulity, ought to put an end to "Professor" Munyon's power of doing mischief in this country. Unfortunately we cannot be sanguine that such a result will be obtained.

